# N-of-1 randomized trials for psychological and health behavior outcomes: a systematic review protocol

**DOI:** 10.1186/s13643-015-0071-x

**Published:** 2015-06-17

**Authors:** Jonathan A. Shaffer, Louis Falzon, Ken Cheung, Karina W. Davidson

**Affiliations:** Department of Medicine, Columbia University Medical Center, 622 West 168th Street, PH9 West, Room 318, New York, NY 10032 USA; Department of Biostatistics, Columbia Mailman School of Public Health, New York, NY USA

**Keywords:** N-of-1, Crossover, Within-subject, Health psychology, Behavioral medicine

## Abstract

**Background:**

Randomized controlled trials are the sine qua non of causal inference; however, heterogeneity of treatment effects for many chronic conditions and for many symptoms often limits their utility. Single-patient studies in which patients select a treatment after trying a randomized sequence of treatments (i.e., multiple crossover trials) offer an alternative to traditional randomized controlled trials by providing scientifically valid results in a practical manner that can be used by patients and their providers to decide upon their personally optimal treatment. Although N-of-1 trials have been used in the medical literature, their use for interventions that consist of psychological or health behavior outcomes is unknown. This systematic review thus aims to describe the interventions and outcomes and assess the quality of N-of-1 trials for psychological or health behavior outcomes.

**Methods/Design:**

Electronic databases (Ovid MEDLINE, EMBASE, CINAHL, PsycINFO, and the six databases in the Cochrane Library) will be searched using all relevant subject headings and free-text terms to represent N-of-1 trials and psychological or behavioral interventions. Full text review and bibliography searching will be conducted. Unpublished studies will be sought by searching trial registries and contacting authors of included studies. Eligibility criteria are the following: population, all human participants for whom N-of-1 trials with psychological or health behavior outcomes have been conducted; interventions, all interventions for which N-of-1 trials have been conducted; comparison, placebo or active treatment control; and outcome, psychological and health behavior outcomes including self-perceived disease severity and psychological phenomena such as mood and affect. Studies that do not contain sufficient trial detail, describe only design or statistical analytic issues in N-of-1 trials without presentation of an N-of-1 trial itself, and/or are not written in the English language are ineligible. Screening, data extraction, and quality assessment will be conducted by two independent reviewers with disagreements resolved through discussion.

**Discussion:**

This systematic review will describe the interventions and outcomes and assess the quality of N-of-1 trials for psychological or health behavior outcomes. The results will clarify the use of this research methodology in the health psychology and behavioral medicine literature and may pave the way for additional N-of-1 trials to be conducted.

**Systematic review registration:**

PROSPERO CRD42015017853

**Electronic supplementary material:**

The online version of this article (doi:10.1186/s13643-015-0071-x) contains supplementary material, which is available to authorized users.

## Background

Despite their commonly lauded role as the gold standard of causal inference and the cornerstone of evidence-based medicine, randomized controlled trials (RCT) often fail to provide evidence for individualized therapeutic decisions. Indeed, heterogeneity of treatment effects (HTE) is evident for many RCTs for many chronic conditions and for many symptoms, such that some RCT-supported treatments can have huge benefits for some patients but can be minimally effective or even harmful for others [1, 2]. Further, traditional, two-arm, parallel group RCTs can be costly to conduct, leaving clinicians and researchers to rely on clinical experience rather than strong experimental evidence [3]. As these conventional RCTs provide only the average treatment effect of an intervention for a group of patients, patients and clinicians need additional information about the effect of a specific treatment for a specific patient for a specific problem [2].

The Evidence-Based Medicine Working Group suggested that N-of-1 trials “provide the strongest evidence for the decisions of patients [4].” N-of-1 trials are single-patient studies in which patients select a treatment after trying a series of treatments (i.e., multiple crossover trials) to determine the relative benefits and harms of each treatment for themselves [1]. They focus on the individual patient by randomizing comparative treatments across time within each patient, rather than randomizing different patients to different comparative treatments as is done in a traditional RCT. Thus, instead of using the results of a conventional or between-patient RCT to choose the best treatment for a patient, the N-of-1 trial methodology can provide scientifically valid results and therefore valuable information in a practical manner that can be used by patients and providers to decide upon a personally optimal treatment and so overcome HTE. In this way, N-of-1 trials are the foundational design for a truly patient-centered comparative effectiveness method. Further, N-of-1 trials are specifically designed to help patients make healthcare decisions that are informed by high-integrity, evidence-based information that is uniquely relevant to their important outcomes and values [5]. In a series of demonstration trials, N-of-1 designs have led to valuable changes in treatment, cessation of treatment, or confirmation of the original treatment [6–10]. For example, in one series of 71 N-of-1 trials for patients with either chronic pain or osteoarthritis, 46 patients (65 %) decided to change their pain medication as a result of the information from the trials, and of the 37 patients using an NSAID or Cox-2 inhibitor drug for pain management before their trials, 12 (32 %) decided that the medication was not helping and stopped it, as a result of their trial results.

Notwithstanding the proliferation of N-of-1 trials in the medical literature, randomized N-of-1 trials have only sporadically been used for treatments that target psychological or health behavior outcomes. Furthermore, among those N-of-1 studies that have considered psychological or health behavior outcomes, information regarding study quality and methods for assessing study quality is limited. This gap in knowledge is peculiar given the abundance of other single participant study designs (e.g., ABA designs, multiple baseline designs, and time-series designs) in the psychology literature and the push to tailor psychological interventions to the individuality of the patient and the singularity of his or her context and condition [11]. It is with these gaps in knowledge in mind that we endeavored to conduct a systematic review of N-of-1 trials of interventions with psychological or health behavior endpoints.

## Methods/Design

### Aims

The proposed review aims to describe the interventions and outcomes and assess the quality of N-of-1 trials for psychological or health behavior outcomes. The reporting of this review will conform to Preferred Reporting Items for Systematic Reviews and Meta-analyses (PRISMA) guidelines [12].

### Eligibility criteria

Population: This review is not limited to a particular population, but will consider all populations for whom N-of-1 trials have been conducted.

Interventions: All medical, psychological, and behavioral interventions for which N-of-1 trials have been conducted (i.e., no restrictions on interventions) will be considered.

Comparator/control: Inclusion criteria require placebo control or an active treatment control.

Outcomes: Psychological and health behavior outcomes will be considered in the following categories:

1. Behavioral—defined as a response that can be conditioned and is objectively observable (e.g., number of steps).
2. Self-perceived disease severity or disease processes—defined as patient ratings of disease severity or disease processes, including but not limited to pain, dyspnea, and gastrointestinal discomfort.
3. Psychological—defined as self-reported affective functioning, including but not limited to depression and anxiety.

### Study design

Only peer-reviewed studies in full text, conference abstract, or doctoral dissertations are eligible for this review. Studies must involve randomization of treatments within blocks or pairs, crossover of interventions, individual patients or series of patients, and single patients as the unit of analysis.

### Exclusion

Studies that do not contain sufficient trial detail, consist primarily of methods and review (e.g., Lillie and colleagues [13] discuss design issues and the analysis of N-of-1 trials but do not report an N-of-1 trial of their own), and/or are not written in English will be ineligible as these will make assessment of study quality impossible.

### Search strategy

Potentially relevant articles will be identified by searching the biomedical electronic databases Ovid MEDLINE, EMBASE, all six databases in The Cochrane Library, CINAHL, and PsycINFO. All relevant subject headings and free-text terms will be used to represent N-of-1 controlled trials and psychological or behavioral interventions, and databases will be searched from inception through the week of planned manuscript submission. Terms for MEDLINE will include the following: n-of-1.tw OR ((individual or single) adj (patient$ or participant$ or subject$)).tw. OR ipd.tw AND exp Behavioral Medicine/OR exp psychotherapy/OR behavio$ adj (change or health or medicine or therap$)).tw OR psychotherap$.tw. OR psycholog$.tw. (see Additional file 1: for full strategies). These terms will be adapted for the other databases. Ongoing studies will also be sought through Clinicaltrials.gov and the WHO International Clinical Trials Registry Platform. Additional records will be identified by scanning the reference lists of relevant studies and reviews, by employing the Related Articles feature in PubMed, and by using the Cited Reference Search in Scopus.

### Search selection process

Two reviewers (JAS, LF) will independently screen titles and abstracts of all the retrieved bibliographic records. Full texts of potentially eligible records passing the title and abstract screening level will be retrieved and examined independently by the two reviewers according to the above mentioned eligibility criteria. Disagreements at both screening levels (title/abstract and full text) will be adjudicated by a third reviewer (KWD). A PRISMA flow chart will outline the study selection process and reasons for exclusions.

### Assessment of study quality

Assessment of study quality will be performed by two reviewers (JAS, LF) according to the Consolidated Standards of Reporting Trials (CONSORT) statement [14] and the CONSORT extension for N-of-1 Trials (CENT) (Table 1) [15]. After determination of a study’s eligibility, the following information will be reviewed and determined to be either reported or not reported: introduction (scientific background and explanation of rationale, specific objectives or hypotheses, rationale for using an N-of-1 approach), trial design characteristics (description of trial design with planned number of periods and duration of each period, individualization of the design for series of participants, important changes to methods after trial commencement, participant eligibility criteria, duration of treatment periods), intervention and outcome characteristics with sufficient detail to allow replication (completely defined primary and secondary endpoints, measurement properties of outcome assessment tools, changes to trial outcomes), allocation characteristics (allocation method, blinding, allocation concealment mechanism, allocation implementation), statistical analytic methods (sample size determination, explanation of interim analyses and stopping guidelines, consideration of carry-over effects, period effects, intra-participant associations, and methods of quantitative synthesis of series data), results (number and sequence of periods completed, number of participants enrolled and assigned to intervention, losses or exclusion of participations after treatment assignment, table showing baseline data, number of periods analyzed, number of trials for which data were synthesized, results for each primary and secondary endpoint per period, estimated effect size and precision, results of any additional analyses), harms or unintended effects for the intervention, and discussion (trial limitations, generalizability, consideration of harms and benefits).

**Table 1:** CENT 2015 checklist: CONSORT 2010 checklist items with modifications or additions for individual or series of N-of-1 trials [15]

Section/topic	Item no.	CONSORT 2010	Item no.	CENT 2015
Title and abstract				
	1a	Identification as a randomized trial in the title	1a	Identification as an “N-of-1 trial” in the title
				For series: identification as “a series of N-of-1 trials” in the title
	1b	Structured summary of trial design, methods, results, and conclusions (for specific guidance see CONSORT for abstracts)	1b	
Introduction				
Background and objectives	2a	Scientific background and explanation of rationale		
	2b	Specific objectives or hypotheses		
			2c	Rationale for using N-of-1 approach
Methods				
Trial design	3a	Description of trial design (such as parallel, factorial) including allocation ratio	3a	Describe trial design, planned number of periods, and duration of each period (including run-in and wash out, if applicable)
				In addition for series: whether and how the design was individualized to each participant and explanation of the series design
	3b	Important changes to methods after trial commencement (such as eligibility criteria), with reasons		
Participant(s)	4a	Eligibility criteria for participants	4a	Diagnosis/disorder, diagnostic criteria, comorbid conditions, and concurrent therapies.
				For series: same as CONSORT item 4a
	4b	Settings and locations where the data were collected		
			4c	Whether the trial(s) represents a research study and if so, whether institutional ethics approval was sought
Interventions	5	The interventions for each group with sufficient details to allow replication, including how and when they were actually administered	5	The interventions for each period with sufficient details to allow replication, including how and when they were actually administered
Outcomes	6a	Completely defined pre-specified primary and secondary outcome measures, including how and when they were assessed	6a.1	Description and measurement properties (validity and reliability) of outcome assessment tools
	6b	Any changes to trial outcomes after the trial commenced, with reasons		
Sample size	7a	How sample size was determined		
	7b	When applicable, explanation of any interim analyses and stopping guidelines		
Randomization				
Sequence generation	8a	Method used to generate the random allocation sequence	8a	Whether the order of treatment periods was randomized, with rationale, and method used to generate allocation sequence
	8b	Type of randomization; details of any restriction (such as blocking and block size)	8b	When applicable, type of randomization; details of any restrictions (e.g., pairs, blocking)
			8c	Full, intended sequence of periods
Allocation concealment mechanism	9	Mechanism used to implement the random allocation sequence (such as sequentially numbered containers), describing any steps taken to conceal the sequence until interventions were assigned		
Implementation	10	Who generated the random allocation sequence, who enrolled participants, and who assigned participants to interventions		
Blinding	11a	If done, who was blinded after assignment to interventions (for example, participants, care providers, those assessing outcomes) and how		
	11b	If relevant, description of the similarity of interventions		
Statistical methods	12a	Statistical methods used to compare groups for primary and secondary outcomes	12a	Methods used to summarize data and compare interventions for primary and secondary outcomes
	12b	Methods for additional analyses, such as subgroup analyses and adjusted analyses	12b	For series: if done, methods of quantitative synthesis of individual trial data, including subgroup analyses, adjusted analyses, and how heterogeneity between participants was assessed, (for specific guidance on reporting syntheses of multiple trials, please consult the PRISMA Statement)
			12c	Statistical methods used to account for carry-over effect, period effects, and intra-subject correlation
Results				
Participant flow (a diagram is strongly recommended)	13a	For each group, the numbers of participants who were randomly assigned, received intended treatment, and were analyzed for the primary outcome	13a.1	Number and sequence of periods completed and any changes from original plan with reasons
			13a.2	For series: the number of participants who were enrolled, assigned to interventions, and analyzed for the primary outcome
	13b	For each group, losses and exclusions after randomization, together with reasons	13c	For series: losses or exclusion of participants after treatment assignment, with reasons, and period in which this occurred, if applicable
Recruitment	14a	Dates defining the periods of recruitment and follow-up		
	14b	Why the trial ended or was stopped	14b	Whether any periods were stopped early and/or whether trial was stopped early, with reason(s)
Baseline data	15	A table showing baseline demographic and clinical characteristics for each group		
Numbers analyzed	16	For each group, number of participants (denominator) included in each analysis and whether the analysis was by original assigned groups	16	For each intervention, number of periods analyzed
				In addition for series: if quantitative synthesis was performed, number of trials for which data were synthesized
Outcomes and estimation	17a	For each primary and secondary outcome, results for each group, and the estimated effect size and its precision (such as 95 % confidence interval)	17a.1	For each primary and secondary outcome, results for each period; an accompanying figure displaying the trial data is recommended
			17a.2	For each primary and secondary outcome, the estimated effect size and its precision (e.g., 95 % confidence interval)
				In addition for series: if quantitative synthesis was performed, group estimates of effect and precision for each primary and secondary outcome
	17b	For binary outcomes, presentation of both absolute and relative effect sizes is recommended		
Ancillary analyses	18	Results of any other analyses performed, including subgroup analyses and adjusted analyses, distinguishing pre-specified from exploratory	18	Results of any other analyses performed, including assessment of carry-over effects, period effects, intra-subject correlation
				In addition for series: if done, results of subgroup or sensitivity analyses
Harms	19	All important harms or unintended effects in each group (for specific guidance see CONSORT for harms)	19	All harms or unintended effects for each intervention (for specific guidance see CONSORT for harms)
Discussion				
Limitations	20	Trial limitations, addressing sources of potential bias, imprecision, and if relevant, multiplicity of analyses		
Generalizability	21	Generalizability (external validity, applicability) of the trial findings		
Interpretation	22	Interpretation consistent with results, balancing benefits and harms, and considering other relevant evidence		
Other information				
Registration	23	Registration number and name of trial registry		
Protocol	24	Where the full trial protocol can be accessed, if available		
Funding	25	Sources of funding and other support (such as supply of drugs), role of funders		

### Extraction of study endpoints

Type of study endpoint (e.g., self-report rating scale, objectively observed behaviors) will be recorded by two independent reviewers (JAS, LF). Extraction data for 20 % of studies will be compared between reviewers to ensure accuracy of data extraction. Review of these outcomes is intended to provide clinicians and researchers with information that may be of use as they design their own N-of-1 trials by allowing them to identify clinical conditions and outcomes that may be particularly amenable to N-of-1 methodology.

## Discussion

This systematic review aims to add to the extant literature by reviewing data concerning N-of-1 randomized trials with psychological and health behavior outcomes. Our review considers a wide variety of interventions and psychological and health behavior outcomes including, but not limited to, complementary and alternative medicine interventions, psychopharmacologic interventions, surgical interventions, behavioral interventions, and psychotherapeutic interventions. The findings will need to be considered alongside the plethora of other single-case designs that have dominated the fields of psychology and medicine to ensure their uniqueness. The findings might therefore serve as a springboard from which other N-of-1 trials could be developed and may minimize the tendency for researchers to miss reporting critical information required to understand the body of evidence available on any one topic.

There are several limitations that will contextualize the findings and generalizability of the proposed review including our a priori decision to not quantitatively aggregate results. We made this decision as we expect disparate outcomes, time periods, and types of interventions across all behavioral and psychological domains. Moreover, we have chosen to exclude reports that contain insufficient trial detail, which limits the scope of this review. This limitation is important as many N-of-1 trials are embedded within larger editorial, review, and other articles, and their exact methodology is likely not ascertainable. Finally, N-of-1 trials themselves may be limited due to the limited resources available to most practitioners. Nonetheless, their advantages over open trials of treatment are obvious, and services to conduct single-patient trials are becoming more available [16].

In conclusion, although parallel-arm, between-person RCTs are the sine qua non of causal inference, there exist additional randomized designs that are useful in certain circumstances, such as when HTE is large and when the symptoms or outcomes can be measured within-person, and the treatment is reversible. N-of-1 trials offer a low-cost means under these circumstances by which to overcome HTE, particularly by allowing for individualization of treatment. Although a previous systematic review has examined N-of-1 trials in the medical literature [1], a review of their use for interventions with psychological and health behavior outcomes in particular is lacking. The proposed review will thus help summarize the available evidence qualitatively and may guide the development of new N-of-1 trials.

## Additional file

Additional file 1:
**Search strategy.** Detailed search strategy proposed for the systematic review.

## Abbreviations

RCT: randomized controlled trial
HTE: heterogeneity of treatment effect
PRISMA: Preferred Reporting Items for Systematic Reviews and Meta-analyses
CONSORT: Consolidated Standards of Reporting Trials
CENT: CONSORT Extension for N-of-1 Trials
