# Supplementary material for: N-of-1 randomized trials for psychological and health behavior outcomes: a systematic review protocol
Source: Syst Rev. 2015 Jun 17;4:87. doi: 10.1186/s13643-015-0071-x (PMC4471921; doi:10.1186/s13643-015-0071-x)
Supplement: Additional file 1: — Search strategy. Detailed search strategy proposed for the systematic review. [file 13643_2015_71_MOESM1_ESM.docx]

Additional file 1: Search Strategy

| **Database** | **Dates searched** | **Number of references retrieved** | **Number after de-duplication** |
| --- | --- | --- | --- |
| **MEDLINE (Ovid)**  and  Ovid MEDLINE(R) In-Process & Other Non-Indexed Citations | 1946 to November Week 3 2014  December 08, 2014 | 1074 | 987 |
| **Cochrane Database of Systematic Reviews**  **CENTRAL**  **DARE**  **HTA Database**  **NHS EED**  All databases are part of *The Cochrane Library*.  [www.thecochranelibrary.com](file:///\\sgbf010.mc.cumc.columbia.edu\DOM_CBCH$\Staff%20folders\Louise\Cochrane\TSC%20Stuff\www.thecochranelibrary.com) | All databases searched December 9, 2014 | 8  139  4  0  0 | 8  13  4  0  0 |
| **EMBASE** | 1980 to 8 Dec 2014 | 200 | 190 |
| **CINAHL (EBSCOHost)** | 1981 to December 9, 2014 | 100 | 86 |
| **PsycInfo (Ovid)** | 1806 to December Week 1 2014 | 333 | 277 |
| **Clinicaltrials.gov** | Searched December 9, 2014 | 30 | 30 |
| **WHO International Clinical Trials Registry Platform** | Searched December 9, 2014 | 0 relevant from 23 retrieved | 0 |
| **Additional Searching**  **PubMed Related Articles** from 7 available initially selected 8 articles  Cited articles from 7 available initially selected 8 articles (Scopus)  References lists of initially selected 8 articles (Scopus and Web of Science used for 6 references; Anderson and dissertation checked manually)  References from Gabler Systematic Review | Searched January 24, 2015 | 11 relevant  48  173  100 | 8  43  28  93 |
| **All databases** |  | **2220** | **1767** |

**MEDLINE**

1. n-of-1.tw.

2. ((individual or single) adj (patient$ or participant$ or subject$)).tw.

3. ipd.tw.

4. or/1-3

5. exp Behavioral Medicine/

6. (health adj2 psycholog$).tw.

7. (behavio$ adj (change or health or medicine or therap$)).tw.

8. exp Health Behavior/

9. exp Behavior Therapy/

10. exp Cognitive Therapy/

11. exp psychotherapy/

12. psychotherap$.tw.

13. psycholog$.tw.

14. or/5-13

15. 4 and 14

16. randomized controlled trial.pt.

17. controlled clinical trial.pt.

18. randomized.ab.

19. placebo.ab.

20. drug therapy.fs.

21. randomly.ab.

22. trial.ab.

23. groups.ab.

24. or/16-23

25. exp animals/ not humans.sh.

26. 24 not 25

27. 15 and 26

**The Cochrane Library**

#1 n-of-1:ti,ab

#2 ((individual or single) next (patient* or participant* or subject*)):ti,ab

#3 ipd:ti,ab

#4 #1 or #2 or #3

#5 MeSH descriptor: [Behavioral Medicine] explode all trees

#6 (health near/2 psycholog*):ti,ab

#7 (behavio* next (change or health or medicine or therap*)):ti,ab

#8 MeSH descriptor: [Health Behavior] explode all trees

#9 MeSH descriptor: [Behavior Therapy] explode all trees

#10 MeSH descriptor: [Cognitive Therapy] explode all trees

#11 MeSH descriptor: [Psychotherapy] explode all trees

#12 psychotherap*:ti,ab

#13 psycholog*:ti,ab

#14 #5 or #6 or #7 or #8 or #9 or #10 or #11 or #12 or #13

#15 #4 and #14

**EMBASE**

#36. #15 AND #35

#35. #30 NOT #34

#34. #31 NOT #33

#33. #31 AND #32

#32. 'human'/de

#31. 'animal'/de OR 'nonhuman'/de OR 'animal experiment'/exp

#30. #16 OR #17 OR #19 OR #21 OR #22 OR #23 OR #24 OR #25 OR #26 OR #27 OR #28 OR #29

#29. 'single blind procedure'/de

#28. 'randomized controlled trial'/de

#27. 'double blind procedure'/de

#26. 'crossover procedure'/de

#25. volunteer*:ab,ti

#24. assign*:ab,ti

#23. single AND next AND blind:ab,ti OR singly AND next AND blind:ab,ti OR single AND next AND

blinded:ab,ti OR singly AND next AND blinded:ab,ti

#22. double AND next AND blind:ab,ti OR doubly AND next AND blind:ab,ti OR double AND next AND

blinded:ab,ti OR doubly AND next AND blinded:ab,ti

#21. placebo*:ab,ti

#19. crossover*:ab,ti OR cross:ab,ti AND over:ab,ti OR cross AND overs:ab,ti OR 'cross over':ab,ti OR 'cross overs':ab,ti

#17. factorial*:ab,ti

#16. random*:ab,ti

#15. #4 AND #14

#14. #5 OR #6 OR #7 OR #8 OR #9 OR #10 OR #11 OR #12 OR #13

#13. psycholog*:ab,ti

#12. psychotherap*:ab,ti

#11. 'psychotherapy'/exp

#10. 'cognitive therapy'/exp

#9. 'behavior therapy'/exp

#8. 'health behavior'/exp

#7. behavio*:ab,ti AND next:ab,ti AND (change:ab,ti OR health:ab,ti OR medicine:ab,ti OR therap*:ab,ti)

#6. health:ab,ti AND n2:ab,ti AND psycholog*:ab,ti

#5. 'behavioral medicine'/de

#4. #1 OR #2 OR #3

#3. ipd:ab,ti

#2. individual:ab,ti OR single:ab,ti AND next:ab,ti AND (patient*:ab,ti OR participant*:ab,ti OR subject*:ab,ti)

#1. 'n of 1':ab,ti

**PsycINFO**

1. n-of-1.tw.

2. ((individual or single) adj (patient$ or participant$ or subject$)).tw.

3. ipd.tw.

4. or/1-3

5. behavioral medicine/

6. (health adj2 psycholog$).tw.

7. (behavio$ adj (change or health or medicine or therap$)).tw.

8. exp health behavior/

9. exp behavior therapy/

10. cognitive therapy/

11. exp Psychotherapy/

12. psychotherap$.tw.

13. psycholog$.tw.

14. or/5-13

15. 4 and 14

16. limit 15 to "2000 treatment outcome/clinical trial"

17. limit 15 to "therapy (best balance of sensitivity and specificity)"

18. 16 or 17

**CINAHL**

S1 TI n-of-1 OR AB n-of-1

S2 TI individual patient* OR AB individual patient* OR TI individual participant* OR AB individual participant* OR TI individual subject* OR AB individual subject* OR TI single patient* OR AB single patient* OR TI single participant* OR AB single participant* OR TI single subject* OR AB single subject*

S3 TI ipd OR AB ipd

S4 S1 OR S2 OR S3

S5 TI health N2 psycholog* OR AB health N2 psycholog*

S6 TI behavio* change OR AB behavio* change OR TI behavio* health OR AB behavio* health OR TI behavio* medicine OR AB behavio* medicine OR TI behavio* therap* OR AB behavio* therap*

S7 (MH "Health Behavior+")

S8 (MH "Behavior Therapy+")

S9 (MH "Cognitive Therapy+")

S10 (MH "Psychotherapy+")

S11 TI psychotherap* OR AB psychotherap*

S12 TI psycholog* OR AB psycholog*

S13 S5 OR S6 OR S7 OR S8 OR S9 OR S10 OR S11 OR S12

S14 S4 AND S13

**Clinicaltrials.gov**

"n-of-1" | Completed | Interventional Studies | Behavioral

"individual patient" | Completed | Interventional Studies | Behavioral

"individual participant" | Completed | Interventional Studies | Behavioral

"individual subject" | Completed | Interventional Studies | Behavioral

"single patient" | Completed | Interventional Studies | Behavioral

"single participant" | Completed | Interventional Studies | Behavioral

“single subject” | Completed | Interventional Studies | Behavioral

**WHO International Clinical Trials Registry Platform**

**Records checked and only relevant completed studies selected**

"n-of-1" in Title

"individual patient" in Title

"individual participant" in Title

"individual subject" in Title

"single patient" in Title

"single participant" in Title

“single subject” in Title
